# Supplementary material for: CT-Based Radiomics to Predict KRAS Mutation in CRC Patients Using a Machine Learning Algorithm: A Retrospective Study
Source: Biomedicines. 2023 Jul 29;11(8):2144. doi: 10.3390/biomedicines11082144 (PMC10452272; doi:10.3390/biomedicines11082144)
Supplement: Supplementary file 1 [file biomedicines-11-02144-s001.zip › biomedicines-2503738-supplementary.pdf]

Supplementary Materials

Table S1. Minimum, median, mean and maximum kappa achieved by each classifier over all the datasets. In bold the best value for each column.

	Kappa (%)			
**Classifier**	**Minimum**	**Median**	**Mean**	**Maximum**
svm(O)	−29.9	10.7	7.4	38.5
elm(O)	−47.4	0.0	−0.4	45.5
lda(O)	−33.2	19.6	**14.0**	42.6
lda(M)	−30.6	17.5	10.2	38.8
dlda(M)	−42.9	2.6	1.0	32.0
mlp(M)	−43.6	4.3	3.4	42.0
ctree(M)	**−25.3**	3.3	3.1	33.0
ridge(M)	−48.1	0.0	0.3	44.9
knn(M)	−34.3	8.3	5.1	28.2
lasso(M)	−61.3	9.9	3.2	38.8
qda(M)	−27.3	0.7	1.3	28.2
nb(M)	−30.0	4.8	6.7	36.4
adaboost(M)	−40.4	6.7	5.1	28.2
logreg(M)	−29.9	9.8	8.9	42.3
lda(P)	−30.6	14.1	9.5	35.4
logreg(P)	−30.6	9.1	7.6	35.4
kfd(P)	−40.0	9.3	7.6	35.4
rf(P)	−28.6	9.7	7.7	38.5
svm(P)	−31.2	10.8	7.6	38.5
adaboost(P)	−37.8	13.6	10.0	**53.7**
mlp(P)	−56.4	5.5	3.4	52.7
sgd(P)	−36.4	9.6	6.3	42.6
ctree(P)	−29.2	7.1	7.4	35.7
gbm(P)	−30.6	9.6	9.6	38.5
avNNet(R)	−44.6	5.9	3.4	42.0
bagging(R)	−33.9	9.8	5.2	24.0
ctree(R)	−26.6	**27.6**	13.1	34.4
knn(R)	−30.6	6.2	7.9	42.0
lda(R)	−30.6	14.1	9.5	35.4
nb(R)	−30.6	2.8	5.0	38.2
neuralnet(R)	−29.9	10.3	9.1	46.6
nnet(R)	−33.2	7.1	6.1	42.9
rpart(R)	−37.1	4.3	2.8	46.0
svm(R)	−44.1	12.0	8.6	39.1

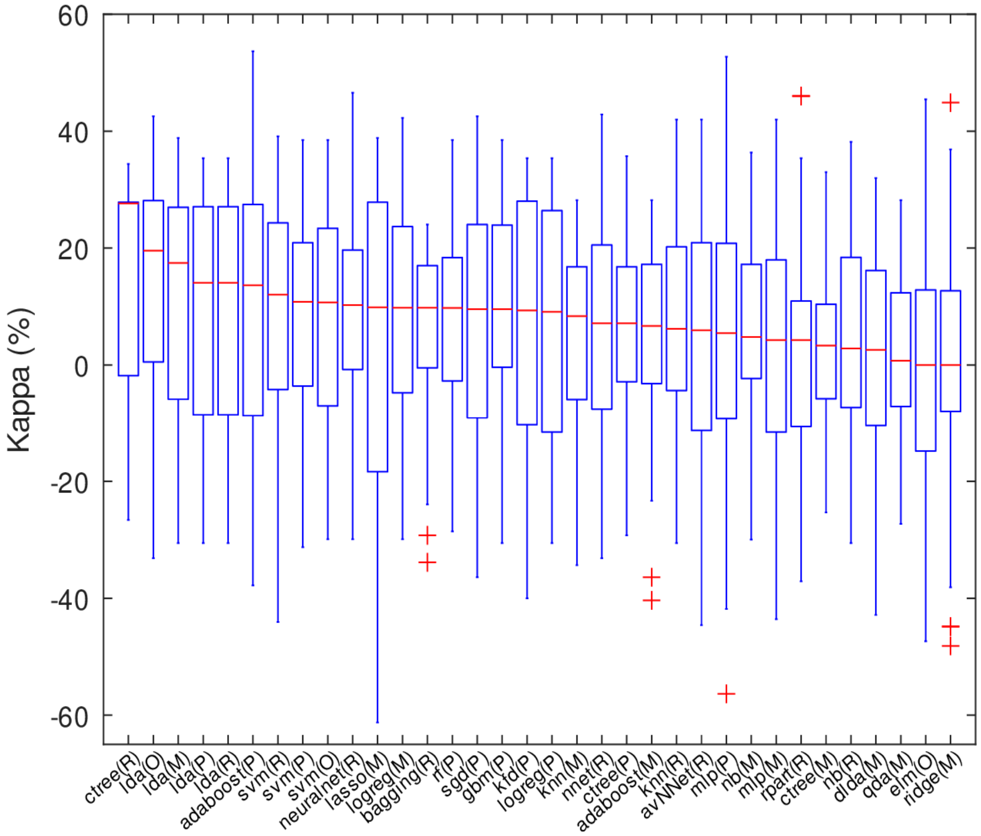

Figure S1. Boxplot of the kappa values achieved by each classifier over all the datasets. The boxes are sorted by decreasing median value (red line inside the box).
